# Weaning order of vasoactive drugs

**DOI:** 10.1186/s13054-018-2218-3

**Published:** 2019-03-13

**Authors:** Vilto Michels, Daisson José Trevisol

**Affiliations:** 1 Postgraduate Program in Health Science, University of Southern Santa Catarina (Unisul), Tubarão, SC Brazil; 2 Clinical Research Center of Nossa Senhora da Conceição Hospital, Tubarão, SC Brazil

In the interesting paper by Jeon et al. [1] the incidence of hypotension while tapering vasopressors in patients recovering from septic shock on concomitant norepinephrine (NE) and vasopressin (AVP) was evaluated. Differing from other studies [2], the authors concluded that the incidence of hypotension was not related to the discontinuation order of the vasopressors, but was related to NE discontinuation. The authors suggested that AVP should be tapered before NE rather than the reverse.

We would like to make some comments on the results.

First, randomization was performed with NE at 0.3 μg/kg/min; then, tapering started at a rate of 0.1 μg/kg/min per hour. In the NE group, the average time to the hypotension onset after initiation of NE tapering was 2.5 h, suggesting that hypotension occurred primarily with NE at low-dose ranges. During NE tapering, commonly, taper rates are slower when the lowest dose ranges are reached due to hypotension [3].

Second, regarding NE administration, total NE time was 57.8 (38.9–88) h and total NE duration before initial tapering was 19.2 h, a very short time for the high hospital mortality found in the study (58.97%). Additionally, total NE tapering time since randomization just before hypotension was 22 h. However, the length of time between the maximum NE dose of 0.70 (0.46–1.20) μg/kg/min and the randomization dose, which could provide further information on tapering and clinical status before randomization, were not mentioned. The number of NE tapering attempts and the volume required during gradual tapering were also not mentioned.

Third, the administration of other drugs may also have affected the outcome. Many patients received dobutamine and low-dose corticosteroid without following a specific protocol.

Fourth, regarding AVP use, AVP infusion was maintained at 0.03 U/min, below its maximum dose of 0.04 U/min in septic shock. AVP has an S-shaped dose-response curve. At intermediate doses, it may have a disproportionately high therapeutic effect variation [4]. Perhaps AVP should be kept at its maximum dose for NE tapering. Also, patients in the AVP group had more frequent pneumonia, greater need for mechanical ventilation, and lower PF ratios than in the NE group, which could have stimulated AVP secretion induced by hypoxemia. This finding also suggests that AVP should be maintained at the maximum dose. Hospital mortality was higher in the AVP group than in the NE group (57.5% versus 34.2%, respectively; *P* = 0.039), which suggests that AVP should be tapered after NE.

## Abbreviations

AVP: Vasopressin
NE: Norepinephrine
